# Axonal protection by Nmnat3 overexpression with involvement of autophagy in optic nerve degeneration

**DOI:** 10.1038/cddis.2013.391

**Published:** 2013-10-17

**Authors:** Y Kitaoka, Y Munemasa, K Kojima, A Hirano, S Ueno, H Takagi

**Affiliations:** 1 Department of Ophthalmology, St. Marianna University School of Medicine, Kawasaki, Kanagawa, Japan

**Keywords:** p62, LC3, Nmnat, autophagy, tumor necrosis factor, glaucoma

## Abstract

Axonal degeneration often leads to the death of neuronal cell bodies. Previous studies demonstrated the crucial role of nicotinamide mononucleotide adenylyltransferase (Nmnat) 1, 2, and 3 in axonal protection. In this study, Nmnat3 immunoreactivity was observed inside axons in the optic nerve. Overexpression of Nmnat3 exerts axonal protection against tumor necrosis factor-induced and intraocular pressure (IOP) elevation-induced optic nerve degeneration. Immunoblot analysis showed that both p62 and microtubule-associated protein light chain 3 (LC3)-II were upregulated in the optic nerve after IOP elevation. Nmnat3 transfection decreased p62 and increased LC3-II in the optic nerve both with and without experimental glaucoma. Electron microscopy showed the existence of autophagic vacuoles in optic nerve axons in the glaucoma, glaucoma+Nmnat3 transfection, and glaucoma+rapamycin groups, although preserved myelin and microtubule structures were noted in the glaucoma+Nmnat3 transfection and glaucoma+rapamycin groups. The axonal-protective effect of Nmnat3 was inhibited by 3-methyladenine, whereas rapamycin exerted axonal protection after IOP elevation. We found that p62 was present in the mitochondria and confirmed substantial colocalization of mitochondrial Nmnat3 and p62 in starved retinal ganglion cell (RGC)-5 cells. Nmnat3 transfection decreased p62 and increased autophagic flux in RGC-5 cells. These results suggest that the axonal-protective effect of Nmnat3 may be involved in autophagy machinery, and that modulation of Nmnat3 and autophagy may lead to potential strategies against degenerative optic nerve disease.

Autophagy is a cellular pathway involved in protein and organelle degradation. p62, which is also called sequestosome 1 (SQSTM1), is a multifunctional protein that interacts with a central component of the autophagy machinery. Autophagy is involved in a cell-protective process and has a role in cell death.^1^ In the retinal neurons, it has been reported that transient increases in microtubule-associated protein light chain 3 (LC3)-II, an autophagic marker, occur in retinal ganglion cells (RGCs) after optic nerve transection.^2^ In a previous study, the protective role of autophagy was demonstrated in RGC-5 cells under conditions of serum deprivation.^2^ The role of autophagy in RGC death is still controversial. For example, autophagy was reported to have a cytoprotective role in RGCs after traumatic injury.^3^ In contrast, it was demonstrated that the inhibition of autophagy resulted in an attenuation of RGC death in a hypertensive glaucoma model.^4^ Those studies focused on RGC body death, although the axonal degeneration pathway in the optic nerve has not been well documented in spite of evidence that the mechanisms of degeneration of neuronal cell bodies and their axons differ.^5, 6, 7^

Axonal degeneration of RGCs is a hallmark of glaucoma. A pattern of localized retinal nerve fiber layer defects in glaucoma patients indicate that axonal degeneration may precede RGC body death in this condition. We previously demonstrated that intravitreal injection of tumor necrosis factor (TNF), which is involved in certain types of glaucoma,^8, 9, 10, 11, 12, 13^ induces progressive optic nerve degeneration with slow RGC body death.^14^ Hypertensive glaucoma rodent models also showed that axon loss precedes RGC body loss.^15^ These observations suggest that both the TNF injection model and hypertensive glaucoma model may be useful in understanding the mechanism of axonal degeneration of RGCs, and the concept of axonal protection can be a powerful tool against neurodegenerative optic nerve disease, such as glaucoma.

It was suggested that nicotinamide mononucleotide adenylyltransferase 1 (Nmnat1), an enzyme predominantly located in the nucleus in the nicotinamide adenine dinucleotide (NAD) biosynthetic pathway, has crucial roles in axonal protection against axotomy in a dorsal root ganglia (DRG) explant culture.^16, 17^ Subcellular localization of Nmnat1 was found to be critical for axonal protection *in vivo*.^18^ We previously found that Nmnat1 is located in the axoplasm of the optic nerve and that depletion of Nmnat1 is involved in optic nerve axonal degeneration.^19^ Other mammalian Nmnat isoforms such as Nmnat2 (located in the Golgi apparatus and cytosol) and Nmnat3 (located in the mitochondria) were also reported to exert axonal protection against axotomy in superior cervical ganglia culture and DRG culture.^20, 21, 22^ The link between Nmnat-mediated protection and autophagy has just started to be reported in dendrite degeneration,^23^ although this link has not been explored in axonal degeneration. As mitochondria has crucial roles in glaucomatous optic neuropathy^24, 25^ and can themselves serve as a part of the autophagosome, we hypothesized that mitochondrial Nmnat3 may alter autophagy machinery.

In the first part of the present study, we evaluated whether Nmnat3 overexpression exerts optic nerve axonal protection using two different axonal degeneration models: the TNF injection model and hypertensive glaucoma model. In the second part, we examined the involvement of autophagy in axonal protection by Nmnat3 in the optic nerve in the hypertensive glaucoma model.

## Results

### Nmnat3 in the optic nerve and retina

**Figure 1:**
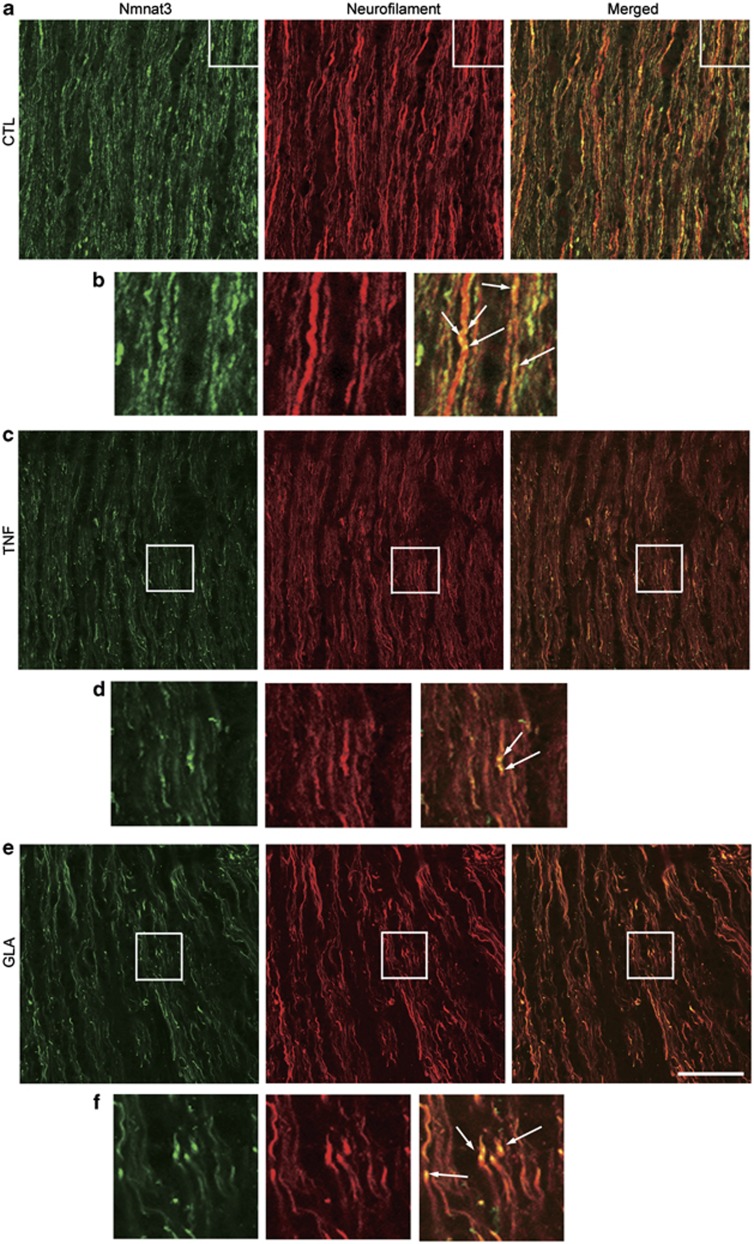
Localization of endogenous Nmnat3 in the optic nerve. (**a**) Double staining for Nmnat3 and neurofilament revealed substantial colocalization in optic nerves in naive samples. (**b**) High-magnification view of the inset in (**a**). Note the Nmnat3-immunopositive dots inside neurofilament-positive fibers (arrows). Similar colocalization of Nmnat3 and neurofilament was observed in the optic nerve 1 week after TNF injection (**c** and **d**) and IOP elevation (**e** and **f**). (**d** and **f**) High-magnification view of the inset in (**c** and **e**). Scale bar =25 *μ*m for (**a**, **c** and **e**) and 8 *μ*m for (**b**, **d** and **f**)

**Figure 2:**
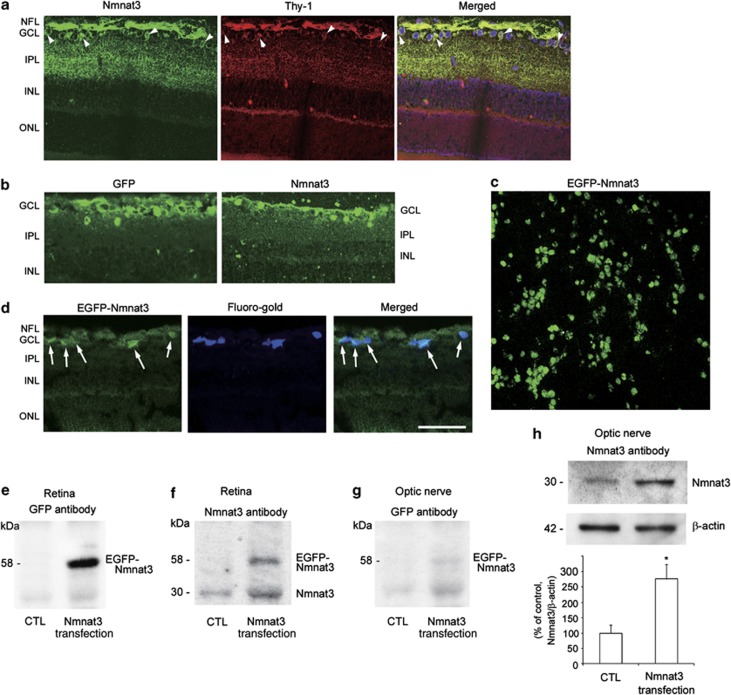
Expression of the EGFP–Nmnat3 in the retina and optic nerve following ELP-mediated transfection. (**a**) Localization of endogenous Nmnat3 in the retina. Double staining for Nmnat3 and Thy-1 revealed some colocalization in cell bodies (arrowheads) and substantial colocalization in nerve fiber layer in naive samples. (**b**) Immunostaining for GFP and Nmnat3 1 week after transfection with EGFP–Nmnat3. (**c**) Flat-mounted retina 1 week after transfection with EGFP–Nmnat3. (**d**) Transverse-sectioned retina 1 week after transfection with EGFP–Nmnat3. Substantial EGFP-positive cells were colocalized with Fluoro-gold-labeled RGCs (arrows). Scale bar=50 *μ*m for (**a**–**d**). (**e**) Immunoblot analysis of GFP expression in the retina 1 week after transfection with EGFP–Nmnat3. (**f**) Immunoblot analysis of Nmnat3 expression in the retina 1 week after transfection with EGFP–Nmnat3. (**g**) Immunoblot analysis of GFP expression in the optic nerve 1 week after transfection with EGFP–Nmnat3. (**h**) Immunoblot analysis of Nmnat3 expression in the optic nerve 1 week after transfection with EGFP–Nmnat3. Immunoblot data are normalized to *β*-actin levels in the same sample. Data are expressed as a percentage of control. Each column represents mean±S.E.M.; *n*=4 per group. *P<0.05 *versus* control

Double-labeling immunohistochemical studies showed substantial colocalization of Nmnat3 and neurofilaments in the laminar portion of the optic nerve in normal rats (Figure 1a). High-magnification photos showed Nmnat3-immunopositive dots inside neurofilament-immunopositive fibers (Figure 1b). These Nmnat3-immunopositive dots were slightly decreased 1 week after TNF injection (Figures 1c and d) or intraocular pressure (IOP) elevation (Figures 1e and f). In the retina, Nmnat3-positive cells were colocalized with Thy-1-positive cells, a marker of RGCs (Figure 2a). Nmnat3 immunoreactivity was also colocalized with Thy-1-immunopositive nerve fibers. Hence, Nmnat3 is localized in both RGC bodies and their axons.

To confirm the transfection of the Nmnat3-enhanced green fluorescent protein (EGFP) plasmid in the retina and optic nerve, immunoblotting using Nmnat3 antibody and GFP antibody was performed 1 week after electroporation (ELP). Immunoblot analysis of Nmnat3 showed that there was a band corresponding to the endogenous Nmnat3 protein in nontransfected retinas (Figure 2f). In transfected retinas, there was a band in the same position and an additional band with higher molecular weight corresponding to EGFP-fused Nmnat3 (Figure 2f). Immunoblot analysis of GFP showed an obvious band corresponding to EGFP-fused Nmnat3 in transfected retinas (Figure 2e). Although immunoblot analysis of GFP showed a faint band corresponding to EGFP-fused Nmnat3 in the optic nerve in transfected eyes (Figure 2g), we confirmed that there was a significant increase in Nmnat3 protein levels in the optic nerve in transfected eyes compared with nontransfected eyes (Figure 2h). GFP immunoreactivity was mainly observed in the RGC layer after transfection with EGFP–Nmnat3 (Figure 2b). In addition, compared with endogenous Nmnat3 (Figure 2a), exogenous Nmnat3 seemed to locate predominantly in cell bodies (Figure 2b). Flat-mounted retinas revealed substantial transfection of the Nmnat3–EGFP plasmid into the cell bodies (Figure 2c). We previously reported that the efficiency of RGC transfection with EGFP plasmids was 40.83–44.1% and that RGCs constituted 79.98–84.74% of all EGFP-transfected cells in the RGC layer.^26, 27^ We also confirmed that EGFP-positive cells were present primarily in RGCs labeled with Fluoro-gold (Fluorochrome, Denver, CO, USA; Figure 2d).

### Effects of overexpression of Nmnat3 on TNF-induced axonal degeneration

**Figure 3:**
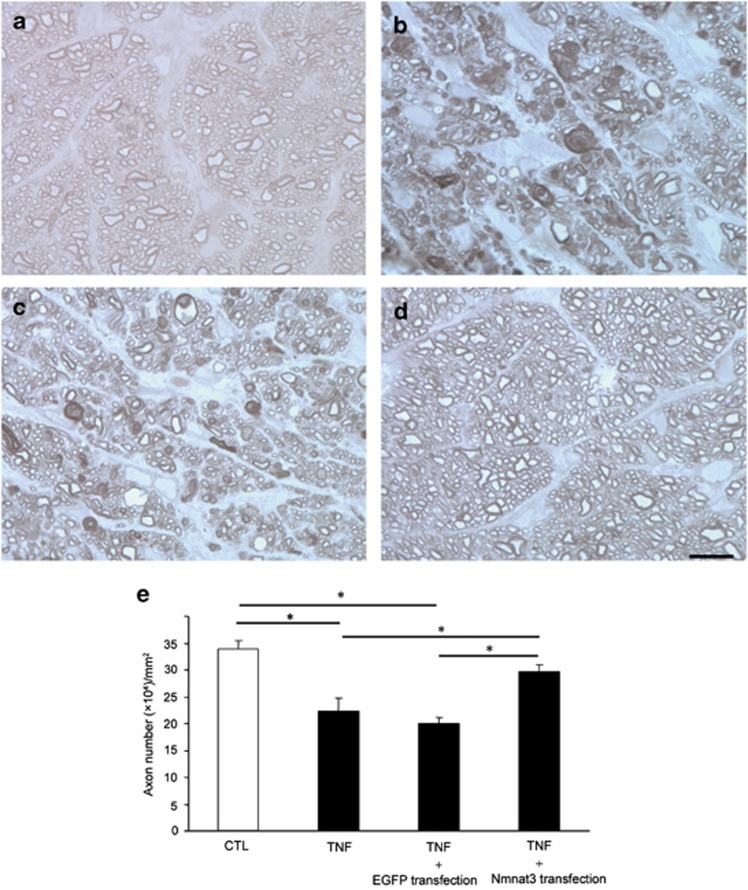
Nmnat3 overexpression prevented TNF-induced axon loss. Light microscopic findings 2 weeks after (**a**) PBS injection, (**b**) 10 ng TNF injection, (**c**) 10 ng TNF injection+EGFP transfection, or (**d**) 10 ng TNF injection+EGFP–Nmnat3 transfection. Scale bar=10 *μ*m (**a**–**d**). (**e**) Effect of Nmnat3 overexpression on axon numbers in the optic nerve. Each column represents mean±S.E.M.; *n*=4–9 per group. *P<0.05

Consistent with our previous report^14, 19^ and in comparison with phosphate-buffered saline (PBS)-treated eyes (Figure 3a), substantial degenerative changes in the axons were noted in nontransfected eyes after TNF injection (Figure 3b) in this study. Similar degenerative changes were also noted in EGFP-transfected eyes (used as negative controls) after TNF injection (Figure 3c). In contrast, EGFP–Nmnat3-transfected eyes showed noticeably attenuated effects with better-preserved nerve fibers (Figure 3d). Quantitative analysis confirmed that there was no significant difference in axon number between nontransfected eyes and EGFP-transfected eyes after TNF injection (*n*=5 for TNF injection with nontransfection, *n*=4 for TNF injection with EGFP transfection; *P*=0.624; Figure 3e). In nontransfected and EGFP-transfected (used as negative controls) eyes after TNF injection, axonal loss was 34 and 41%, respectively, compared with controls. Overexpression of Nmnat3 exerted a significant protective effect against TNF-induced axonal loss (*n*=9; *P*<0.05 *versus* TNF injection with nontransfection, *P*<0.05 *versus* TNF injection with EGFP transfection; Figure 3e). EGFP–Nmnat3-transfected eyes showed 68.5% axonal protection compared with EGFP-transfected eyes after TNF injection (Figure 3e).

### IOP elevation

**Figure 4:**
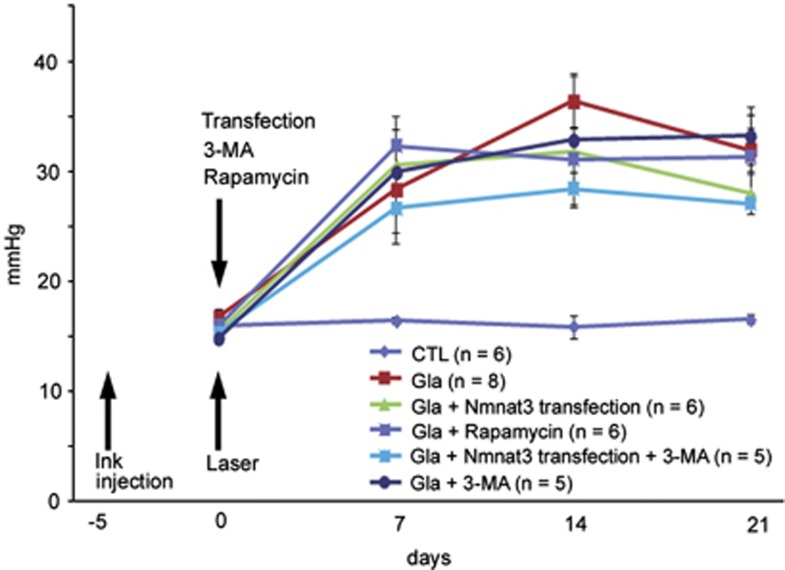
Time course of IOP changes in the control (*n*=6), experimental glaucoma (*n*=8), glaucoma+Nmnat3 transfection (*n*=6), glaucoma+Nmnat3 transfection+3-MA (*n*=5), glaucoma+rapamycin (*n*=6), and glaucoma+3-MA (*n*=5) groups. Significant differences in IOP values were observed in laser-treated animals compared with the control group at 7, 14, and 21 days (*P*<0.05 or 0.005). No significant differences in IOP values were observed between the experimental glaucoma and glaucoma+rapamycin groups, between the glaucoma and glaucoma+3-MA groups, or between the glaucoma+Nmnat3 transfection and glaucoma+Nmnat3 transfection+3-MA groups at any time point

The IOP of laser-treated eyes was compared with that of the contralateral eyes, which served as controls. Significant differences in IOP in the laser treatment groups compared with the control group were observed 1, 2, and 3 weeks after laser treatment (Figure 4). No significant difference in IOP was observed between the glaucoma group and glaucoma+rapamycin, or glaucoma+3-methyladenine (3-MA) group. In addition, no significant difference in IOP was observed between the glaucoma+Nmnat3 transfection group and glaucoma+Nmnat3 transfection+3-MA group.

### Effects of overexpression of Nmnat3, 3-MA, and rapamycin on axonal degeneration after IOP elevation

**Figure 5:**
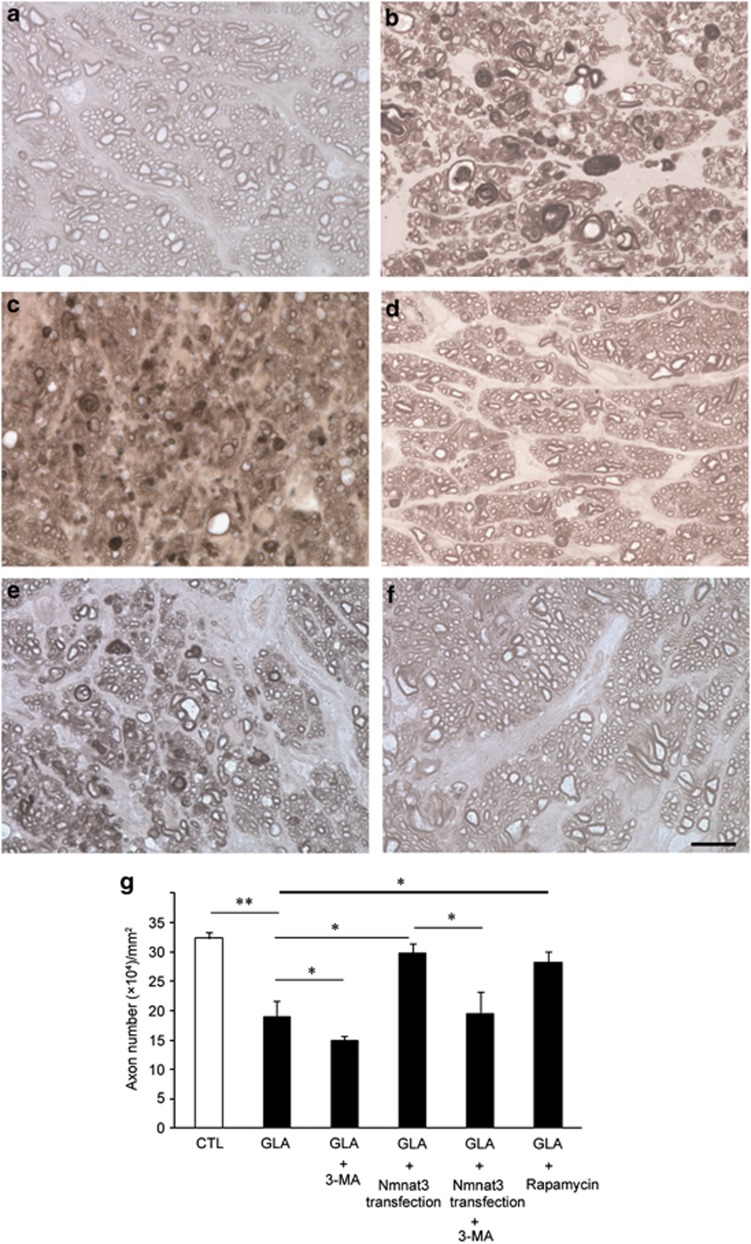
Nmnat3 overexpression prevented axon loss in experimental glaucoma. Light microscopic findings after 3 weeks in the (**a**) control, (**b**) IOP elevation (glaucoma), (**c**) glaucoma+3-MA, (**d**) glaucoma+Nmnat3 transfection, (**e**) glaucoma+Nmnat3 transfection+3-MA, or (**f**) glaucoma+rapamycin groups. Scale bar=10 *μ*m (**a**–**f**). (**g**) Effect of Nmnat3 overexpression on axon numbers in the optic nerve. Each column represents mean± S.E.M.; *n*=5–8 per group. *P<0.05; **P<0.005

Consistent with our previous report^25^ and in comparison with control eyes (Figure 5a), noticeable degenerative changes in axons were observed 3 weeks after IOP elevation (Figure 5b). 3-MA, an autophagy inhibitor, exaggerated axonal degeneration induced by IOP elevation (Figure 5c). In contrast, EGFP–Nmnat3-transfected eyes showed substantially attenuated degenerative effects (Figure 5d). Quantitative analysis confirmed that overexpression of Nmnat3 exerted a significant protective effect against axonal loss induced by IOP elevation (*P*<0.05 *versus* experimental glaucoma, Figure 5g). This protective effect was significantly inhibited by 3-MA, an autophagy inhibitor (*P*<0.05 *versus* experimental glaucoma+Nmnat3 transfection, Figures 5e and g). On the other hand, rapamycin-treated eyes showed noticeably attenuated effects after IOP elevation (Figure 5f), and this protective effect was statistically significant compared with the experimental glaucoma group (*P*<0.05 *versus* experimental glaucoma; Figure 5g).

### Electron microscopy findings after IOP elevation

**Figure 6:**
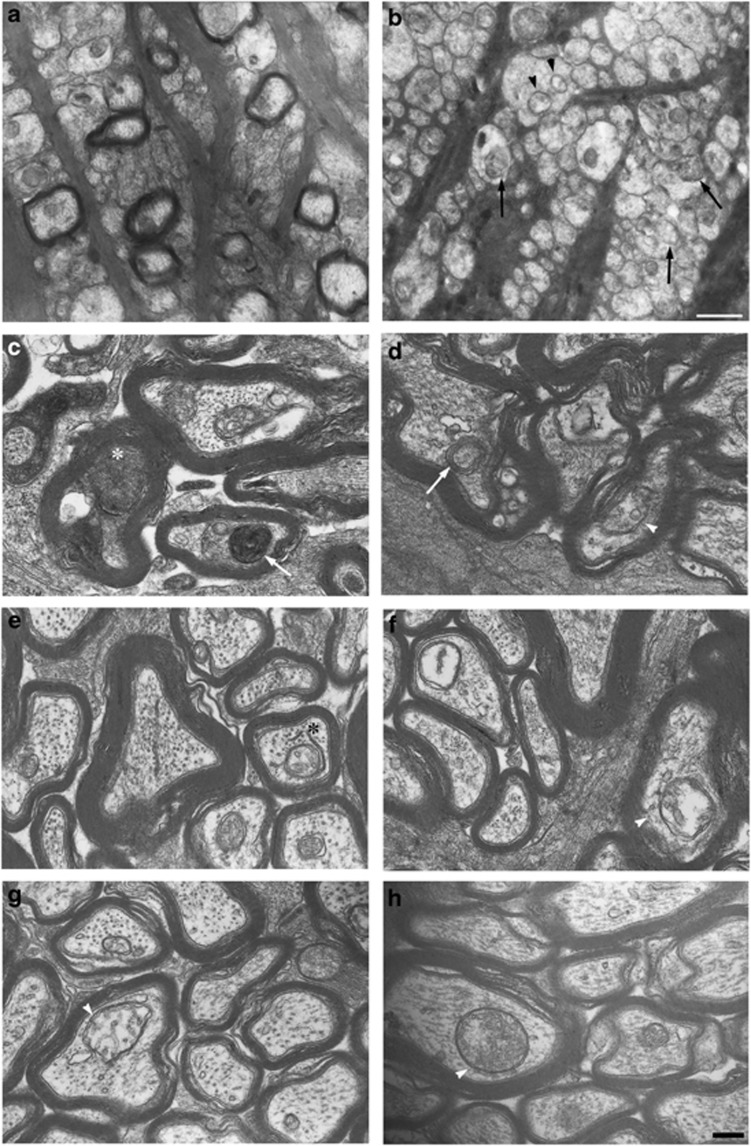
Electron microscopy findings 3 weeks after IOP elevation. Laminar portion in the control (**a**) and experimental glaucoma (**b**) groups. Abnormal mitochondria (black arrowheads) and autophagic vacuoles (black arrows) were noted in unmyelinated axons in experimental glaucoma (**b**). Myelinated portion in the experimental glaucoma (**c** and **d**), glaucoma+Nmnat3 transfection (**e** and **f**), and glaucoma+rapamycin (**g** and **h**) groups. Degenerative changes such as neurofilament accumulation (white asterisk) were noted in experimental glaucoma (**c**). The formation of multilamellar bodies is characteristic of autophagic vacuoles (white arrows; **c** an **d**). Preserved myelin and microtubule structures were noted in glaucoma+Nmnat3 transfection (**e** and **f**) and glaucoma+rapamycin (**g** and **h**) groups. A phagophore was observed in the glaucoma+Nmnat3 transfection (black asterisk; **e**) group. Autophagic vacuoles (white arrowheads) were observed in the glaucoma (**d**), glaucoma+Nmnat3 transfection (**f**), and glaucoma+rapamycin (**g** and **h**) groups. Scale bar=1 *μ*m (**a** and **b**) and 200 nm (**c**–**h**)

Numerous mitochondria were observed inside axons of the laminar portion in the control groups (Figure 6a). On the other hand, abnormal mitochondria and autophagic vacuoles were observed in unmyelinated axons of the laminar portion 3 weeks after IOP elevation (Figure 6b). At higher magnification, consistent with the light microscopy findings showing that noticeable degenerative changes were apparent, degenerative changes such as neurofilament accumulation were observed in the experimental glaucoma groups in the myelinated portion (Figure 6c). Autophagic vacuoles were observed in the glaucoma (Figure 6d), glaucoma+Nmnat3 transfection (Figure 6f), and glaucoma+rapamycin groups (Figures 6g and h). In spite of the appearance of autophagic vacuoles in these groups, degenerative changes were only noted in the glaucoma groups (Figures 6c and d). In the glaucoma+Nmnat3 transfection (Figures 6e and f) and glaucoma+rapamycin groups (Figures 6g and h), myelin and microtubule structures were well preserved, and no apparent degenerative changes were observed.

### Effects of IOP elevation and overexpression of Nmnat3, 3-MA, and rapamycin on p62 and LC3-II protein levels in optic nerves

**Figure 7:**
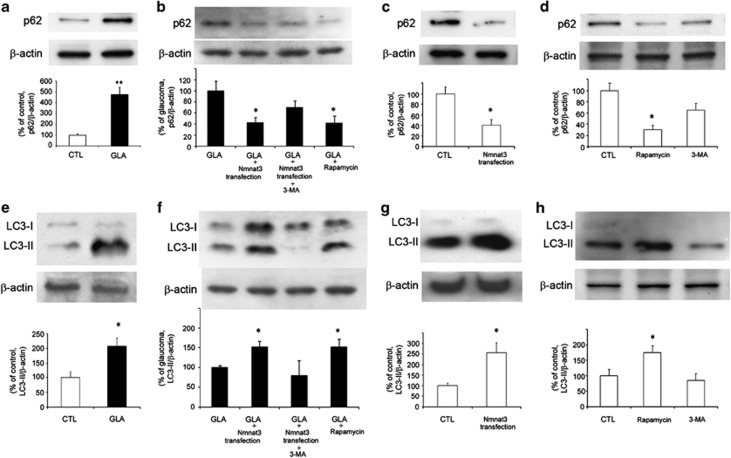
p62 and LC3-II protein levels in optic nerves. Immunoblot data are normalized to *β*-actin levels in the same sample. All data are expressed as a percentage of control except (**b** and **f**). Each column represents mean±S.E.M. (**a**) Immunoblotting for p62 1 week after IOP elevation. *n*=6 per group. **P<0.005 *versus* control. (**b**) Immunoblotting for p62 1 week after IOP elevation. Data are expressed as a percentage of glaucoma. *n*=4 per group. *P<0.05 *versus* glaucoma. (**c**) Immunoblotting for p62 1 week after Nmnat3 transfection. *n*=4 per group. *P<0.05 *versus* control. (**d**) Immunoblotting for p62 1 week after intravitreal injection of rapamycin or 3-MA. *n*=4 per group. *P<0.05 *versus* control. (**e**) Immunoblotting for LC3-II 1 week after IOP elevation. *n*=4 per group. *P<0.05 *versus* control. (**f**) Immunoblotting for LC3-II 1 week after IOP elevation. Data are expressed as a percentage of glaucoma. *n*=4 per group. *P<0.05 *versus* glaucoma. (**g**) Immunoblotting for LC3-II 1 week after Nmnat3 transfection. *n*=5 per group. *P<0.05 *versus* control. (**h**) Immunoblotting for LC3-II 1 week after intravitreal injection of rapamycin or 3-MA. *n*=4 per group. *P<0.05 *versus* control

To address the involvement of autophagy in axonal protection, we examined the changes in p62, a multifunctional protein that interacts with a central component of the autophagy machinery, and LC3-II, an autophagic marker, in the optic nerve. There was a substantial increase in p62 protein levels in the optic nerve samples 1 week after IOP elevation (Figure 7a). This increase was significantly inhibited by Nmnat3 transfection and rapamycin (Figure 7b). Nmnat3 transfection alone significantly decreased p62 protein levels in the optic nerve compared with the basal level (Figure 7c). Treatment with rapamycin also significantly decreased p62 protein levels compared with the basal level (Figure 7d).

There was an increase in LC3-II protein levels in the optic nerve samples 1 week after IOP elevation (Figure 7e). Nmnat3 transfection and rapamycin resulted in further elevation of LC3-II protein levels, but 3-MA did not, in the optic nerve in the glaucoma group (Figure 7f). Nmnat3 transfection alone significantly increased LC3-II protein levels in the optic nerve compared with the basal level (Figure 7g). Rapamycin treatment also significantly increased LC3-II protein levels in the optic nerve compared with the basal level (Figure 7h).

### Expression of p62 in RGC-5 cells and autophagic flux

**Figure 8:**
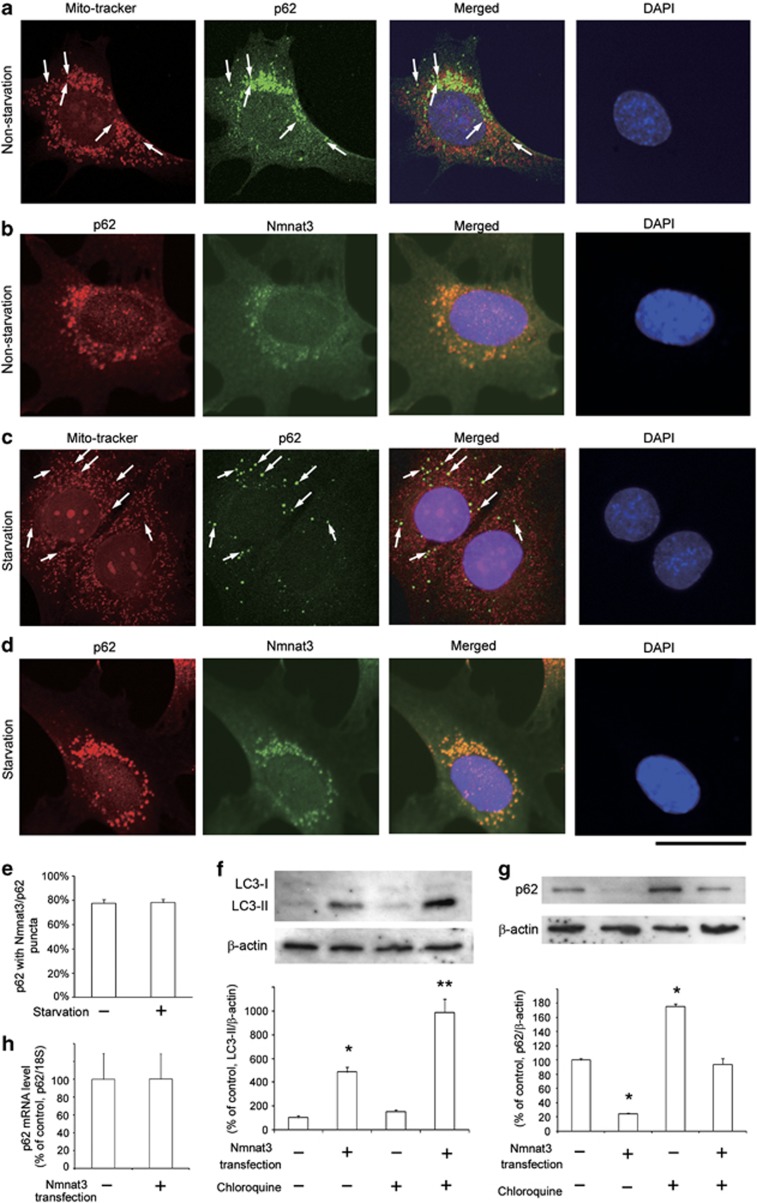
Subcellular localization of p62 and Nmnat3 in non-starved and starved RGC-5 cells and effect of Nmnat3 transfection on the LC3 turnover assay. p62 was colocalized with MitoTracker Red, a marker of mitochondria in non-starved (**a**) and starved (**c**) RGC-5 cells. p62 was colocalized with Nmnat3 in non-starved (**b**) and starved (**d**) RGC-5 cells. Scale bar=25 *μ*m. (**e**) Quantification of Nmnat3 positivity (%) of the p62 puncta in non-starved and starved RGC-5 cells. Data represent mean±S.E.M. of 22 images. (**f**) LC3 turnover assay. The difference in LC3-II levels between RGC-5 cells with and without chloroquine was compared under conditions of nontransfection and transfection with Nmnat3. *n*=3 per group. *P<0.05 *versus* control; **P<0.05 *versus* transfection. (**g**) Immunoblotting for p62 24 h after Nmnat3 transfection in RGC-5 cells with and without chloroquine. *n*=3 per group. *P<0.05 *versus* control. (**h**) p62 mRNA levels 24 h after Nmnat3 transfection in RGC-5 cells. *n*=4 per group

A previous study demonstrated that LC3 in starved RGC-5 cells showed a punctuated staining pattern, suggesting the formation of autophagosomes.^2^ In the present study, we examined the subcellular localization of p62 in non-starved and starved RGC-5 cells. Some p62-immunopositive dots were colocalized with MitoTracker Red (Invitrogen, Carlsbad, CA, USA) in non-starved RGC-5 cells, and this colocalization was more prominent in starved RGC-5 cells, indicating that p62 was present in mitochondria (Figures 8a and c). This finding is consistent with a recent study demonstrating that p62 was present in the mitochondria in mouse embryonic fibroblasts.^28^ We confirmed substantial colocalization of the mitochondrial Nmnat3 and p62 in non-starved and starved RGC-5 cells (Figures 8b–e). Next, we performed the LC3 turnover assay in RGC-5 cells. The difference in LC3-II levels in the presence and absence of chloroquine was greater with Nmnat3 transfection (Figure 8f), indicating that autophagic flux is increased with Nmnat3 transfection. On the other hand, in the optic nerve, no difference in LC3-II levels in the presence and absence of chloroquine was observed in the glaucoma group compared with the control group (Supplementary Figure S1), suggesting that there was autophagic flux blocking in the optic nerve after IOP elevation. In addition, Nmnat3 transfection significantly decreased p62 protein levels in RGC-5 cells (Figure 8g). In these experiments, the effects of chloroquine on the accumulation of LC3-II and p62 were observed when compared with the basal levels. For example, chloroquine increased LC3-II up to 152% in RGC-5 cells (*P*=0.275; Figure 8f), increased LC3-II up to 162% in optic nerves (*P*<0.05; Supplementary Figure S1), and increased p62 up to 175% in RGC-5 cells (*P*<0.05; Figure 8g). As it has been reported that p62 protein levels might also change because of its regulation at the transcription level,^29^ we examined p62 mRNA levels after Nmnat3 transfection in RGC-5 cells. We found that there was no significant difference between the control and Nmnat3 transfection groups in RGC-5 cells (Figure 8h).

## Discussion

NAD, a key intermediate in cellular energy homeostasis, and its associated biosynthetic pathway have been suggested to have a crucial role in axonal protection against mechanical and chemical injury. In the current study, we examined the localization of Nmnat3 in the retina and optic nerve and evaluated whether Nmnat3 overexpression exerts axonal protection against optic nerve degeneration induced by TNF and IOP elevation. We found that autophagy machinery is involved in axonal degeneration after IOP elevation and in protective processes of exogenous Nmnat3.

First, Nmnat3 was present in the optic nerve axon. Our immunolabeling study showed that Nmnat3 localizes inside axon nerve fibers, and their dot-like pattern is consistent with a previous demonstration that Nmnat3 locates in mitochondria.^30^ In addition, we observed that Nmnat3 is present in RGC bodies and nerve fibers in retina. In RGC bodies and their axons in the unmyelinated portion, the number of mitochondria was reported to be higher than that in myelinated axons, suggesting that mitochondria serve the high-energy requirements for conduction in unmyelinated axons.^31^ Slight decreases in mitochondrial Nmnat3 inside axon fibers after TNF injection and IOP elevation are in accordance with a previous finding that mitochondria are decreased in the optic nerve after IOP elevation.^25^ We next examined the efficacy of the transfection of the Nmnat3–EGFP plasmid into the retina and optic nerve. We previously demonstrated that, although transfected cells were not specifically RGCs, most transfected cells in the ganglion cell layer were RGCs.^26, 27^ In the present study, we observed substantial transfection of the Nmnat3–EGFP plasmid into RGC bodies and a significant increase in Nmnat3 protein levels in the optic nerve. Thus, it is possible that transfected RGCs can upregulate the Nmnat3 protein, thereby leading to increased Nmnat3 protein levels in the optic nerve. These findings are consistent with previous results demonstrating that transfection of thioredoxin 2 (Trx2), which is also located in the mitochondria, leads to an increase in Trx2 protein levels in the optic nerve.^25^

Overexpression of Nmnat3 exerted a significant protective effect against TNF-induced axonal loss. This axon-protective effect of Nmnat3 is consistent with other published reports demonstrating that overexpression of Nmnat3 provided strong axonal protection after transection in DRG neurons.^22^ Moreover, overexpression of Nmnat3 was reported to protect against rotenone-mediated (mitochondrial dysfunction) axonal degeneration and to delay axonal degeneration induced by treatment with the oxidant H_2_O_2_ in DRG neurons.^32^ Further, it was reported that transgenic mice overexpressing NMNAT3 had a significant number of preserved axons in injured sciatic nerves, whereas wild-type mice had mostly degraded axons in injured nerves.^33^ Thus, increased Nmnat3 levels both *in vitro* and *in vivo* when using transgenic mice and local overexpression as in the present study appear to exert axonal protection. We postulate that overexpression of Nmnat3 in mitochondria may lead to an increase in NAD^+^ in mitochondria. Whether these mitochondria can translocate and contribute to axonal protection remains to be elucidated.

Overexpression of Nmnat3 exerted a significant protective effect against axonal loss after IOP elevation. This protective effect was inhibited by 3-MA, an autophagy inhibitor, consistent with the results of a previous study demonstrating that exposure to 3-MA significantly reduced cell viability under starvation in RGC-5 cells.^34^ Taken together with our observation of autophagic vacuoles in the electron microscopy findings, these results suggest that the protective effect of Nmnat3 may be involved in the autophagy machinery in optic nerve axons. In addition, rapamycin, an autophagy inducer, exerted a significant protective effect against axonal loss after IOP elevation, consistent with the results of a recent study demonstrating that decreased Brn-3a-immunopositive RGCs in flat-mounted retinas after optic nerve transection were significantly increased by rapamycin.^3^ That study showed that rapamycin decreased intracellular reactive oxygen species (ROS) production and increased cell viability and that 3-MA increased ROS production and reduced cell viability in RGC-5 cells.^3^ These findings are also consistent with the present demonstration that 3-MA enhanced axonal degeneration after IOP elevation. However, this finding is contradictory to the results of another study demonstrating that decreased 4′,6-diamidino-2-phenylindole (DAPI)-positive cells after IOP elevation in the ganglion cell layer were significantly increased by 3-MA.^4^

A recent study has demonstrated that overexpression of p62 promotes apoptosis with the activation of caspase-8, whereas knockdown of p62 reduces cell death.^35^ p62 is normally degraded by the lysosomal proteases through the interaction with LC3-II.^36^ In the present study, there were increases in p62 and LC3-II in the optic nerve after IOP elevation. Considering these results and taken together with the finding that chloroquine did not increase in LC3-II in the optic nerve after IOP elevation, it is possible that autophagic flux blocking may be involved in these processes. It is interesting to note that diminished autophagic flux in trabecular meshwork cells has been suggested to contribute to the pathogenesis of glaucoma.^37^ Thus, these findings suggest that impaired autophagic flux may be an important manifestation not only in trabecular meshwork cells but also in optic nerves in glaucoma. It was demonstrated that under pathological conditions, there is a constitutively high level of p62, thereby leading to the accumulation of damaged mitochondria and subsequent ROS production.^38^ Several studies proposed molecular mechanisms of Nmnat-regulated axonal protection. For example, a recent study has demonstrated that the highwire ubiquitin ligase is a critical regulator of Nmnat and may have a central role in regulating the ability of a neuron to regenerate its connection.^39^ Another study suggested that the molecular chaperones are the key factors in Nmnat-regulated axonal-protective functions.^40^ Here, we found a direct relationship between Nmnat3 and p62 at the subcellular level. It is particularly important to note that Nmnat3 transfection increased autophagic flux and decreased p62 protein levels in RGC-5 cells. This increase in autophagic flux represents autophagy induction rather than a block in downstream steps, such as autophagolysosomal maturation.^41^ It was shown that the activation of autophagy increased protein levels of LC3-II and Beclin1 and decreased p62 in neuroblastoma SH-SY5Y cells.^42^ In addition, we observed that Nmnat3 transfection decreased p62 protein levels and increased LC3-II protein levels in the optic nerve both with and without glaucoma. Therefore, it is possible that the protective effect of Nmnat3 is associated with its enhancement of outgoing p62 flux and its induction of LC3-II. We also found that rapamycin increased LC3-II levels and decreased p62 levels in the optic nerve. These findings are consistent with those of a previous study demonstrating that LiCl, an autophagy inducer, increased the expression of LC3-II under hypoxic stress and decreased the expression of p62 under normoxia and hypoxic stress in the neuronal cell culture.^43^

In conclusion, the present study showed the presence of Nmnat3 in the optic nerve axon. Overexpression of Nmnat3 exerts axonal protection against TNF-induced and IOP elevation-induced optic nerve degeneration. Transfection of Nmnat3 can alter the autophagy machinery, and the protective effect of Nmnat3 may be involved in decreased p62 and increased LC3-II levels in optic nerve degeneration.

## Materials and Methods

### Animals

Experiments were performed on 50- to 55-day-old male Wistar rats. All studies were conducted according to the Association for Research in Vision and Ophthalmology statement for the Use of Animals in Ophthalmic and Vision Research and were approved by the Ethics Committee of the Institute of Experimental Animals of St. Marianna University Graduate School of Medicine. The animals were housed in controlled conditions, with temperature at 23±1 °C, humidity at 55±5%, and light from 0600 to 1800 hours.

### Administration of TNF

Intravitreal injection of TNF (Sigma-Aldrich, St. Louis, MO, USA) was performed as described previously.^14, 19^ Briefly, rats were anesthetized with an intramuscular injection of a mixture of ketamine-xylazine (10 and 4 mg/kg, respectively). A single 2-*μ*l injection of 10 ng TNF in 0.01 M PBS, pH 7.40, was administered intravitreally into the right eye of an animal under a microscope to avoid lens injury. PBS alone was administered into the contralateral left eye as a control. The rats were euthanized 1 or 2 weeks after the intravitreal injections with an intraperitoneal overdose of sodium pentobarbital, followed by enucleation of the eye.

### Hypertensive glaucoma model and IOP measurements

The rats were maintained for at least 1 week before IOP measurement. A rat glaucoma model was generated as described previously.^25^ Briefly, rats were anesthetized with an intramuscular injection of a mixture of ketamine-xylazine (10 and 4 mg/kg, respectively). A single 10-*μ*l injection of 35% India ink (Becton Dickinson, Sparks, MD, USA) in 0.01 M PBS was administered intracamerally to the right eye of an animal after removing an equal volume of aqueous humor under a microscope to avoid lens injury. A dark circumferential band at the limbus was noted in the trabecular meshwork because of the aggregation of carbon particles 5 days after injection. Approximately 200 laser burns were delivered *ab externo* to the pigmented trabecular band at an argon laser setting of 200 *μ*m in diameter, 150–200 mW, for 0.2-s durations (Ultima argon laser 2000 SE, Coherent, Palo Alto, CA, USA). The contralateral left eyes were used as controls. In the rapamycin and 3-MA treatment group, a 2-*μ*l injection of 1 mM rapamycin (LC Laboratories, Woburn, MA, USA) or 60 mM 3-MA (Sigma-Aldrich) in dimethyl sulfoxide (DMSO) was administered intravitreally 6 h before glaucoma induction.

Dark-phase IOP was monitored once a week with a portable tonometer (Tonolab, Tiolat, Helsinki, Finland). IOP was measured in animals in the awake state 1 h after initiation of the dark phase. Following topical instillation of 0.4% oxybuprocaine hydrochloride (Santen, Osaka, Japan), the tonometer was gently and briefly applied to the cornea and the IOP reading was recorded. Five consecutive readings were taken. After eliminating the minimum and maximum measurements, the remaining three readings were averaged. The rats were euthanized with an intraperitoneal overdose of sodium pentobarbital 1 or 3 weeks after the intravitreal injections, followed by enucleation of the eye. In the chloroquine treatment group, chloroquine (60 mg/kg; Sigma-Aldrich)^44^ was administered intraperitoneally 12 h before enucleation of the eye.

### Immunohistochemistry

Twelve rats were used for immunohistochemical study. Eyes from naive, 1week after TNF injection, or 1 week after IOP elevation samples were fixed by immersion in 10% neutral-buffered formalin for 24 h, dehydrated, embedded in paraffin, and sectioned (4 *μ*m thick) through the optic disc. Deparaffinized sections were incubated with 1% bovine serum and then reacted with primary antibodies against Nmnat3 (1 : 100; ProSci Incorporated, Poway, CA, USA), neurofilament-L (a marker of neurons; 1 : 100; DAKO Corporation, Carpinteria, CA, USA), or Thy-1 (a marker of RGCs; 1 : 100; Santa Cruz Biotechnology, Inc., Santa Cruz, CA, USA) diluted in 1% bovine serum overnight at 4 °C. Sections were then exposed to the following secondary antibodies: FITC-labeled anti-goat antibody (1 : 100; Cappel, MP Biomedicals, LLC., Solon, OH, USA) or rhodamine-labeled anti-mouse antibody (1 : 100; Cappel, MP Biomedicals, LLC.). For retinal immunostaining, the samples were counterstained with DAPI (Vectashield with DAPI, Vector Laboratories, Inc., Burlingame, CA, USA). Negative controls were performed by replacing the primary antibody with PBS or serum.

### Immunoblot analysis

Seventy rats were used for immunoblot analysis as described previously.^45^ Briefly, 1 week after IOP elevation, ELP, or intravitreal injection, optic nerves (4-mm in length) were collected, homogenized, and then centrifuged at 15 000 × *g* for 15 min at 4 °C. Two optic nerve specimens were pooled for one sample. Protein concentrations were determined using the Bio-Rad Protein Assay kit (Bio-Rad, Hercules, CA, USA). Protein samples (5 *μ*g per lane for the optic nerve) were subjected to SDS-PAGE on gels (Bio-Rad) and transferred to PVDF membranes (Immobilon-P, Millipore, Billerica, MA, USA). Membranes were blocked with Tris-buffered saline (TBS)-0.1% Tween-20 containing 5% skim milk. Membranes were first reacted with anti-Nmnat3 antibody (1 : 200; ProSci Incorporated), anti-p62 antibody (1 : 200; Medical & Biological Laboratories Co., Nagoya, Japan), anti-LC3 (1 : 200; Medical & Biological Laboratories Co.), anti-GFP (1 : 200; Millipore), or anti-*β*-actin antibody (1 : 500; Sigma-Aldrich) in TBS containing 5% skim milk. Membranes were then sequentially exposed to peroxidase-labeled anti-goat IgG antibody (Cappel, MP Biomedicals, LLC.), peroxidase-labeled anti-rabbit IgG antibody (Cappel, MP Biomedicals, LLC.), or peroxidase-labeled anti-mouse IgG antibody (Cappel, MP Biomedicals, LLC.) diluted 1 : 5000 in Tween-20 in TBS. Western blots were visualized with an ECL detection system (Amersham ECL Prime Western Blotting Detection Reagents, GE Healthcare, Buckinghamshire, UK).

### Gene transfer to the retina and optic nerve via *in vivo* ELP

The cDNA encoding rat Nmnat3 was obtained by RT-PCR from total RNA extracted from rat retina with the primers 5′-CTTCGAATTCTCACCATGAAGAACCGAATCCCCGTG-3′ and 5′-GATGGATCCGGCAGTGCCGCAGAGGCTGATG-3′ (GenBank accession number NM_001013224), and then inserted into a recombinant plasmid of EGFP-N3. Nmnat3-expressing plasmid DNA was delivered to RGCs via ELP, as described previously.^25, 26^ Briefly, rats were anesthetized with an intramuscular injection of a mixture of ketamine-xylazine (10 and 4 mg/kg, respectively). Plasmid DNA (4 *μ*l; 2.5 *μ*g/*μ*l) was injected into the vitreous cavity with a 34-gauge needle 0.5 mm posterior to the limbus under a microscope. After 10 min, the cathodal electrode was placed on the cornea, and an 18-gauge needle with an attached anodal electrode was inserted subcutaneously in the middle of the forehead. Electric pulses were generated by a pulse generator (CUY21SC, Nepa Gene, Chiba, Japan). Parameters were as follows: electric field strength of 6 V/cm; pulse duration of 99 ms; and stimulation pattern of five pulses at a frequency of 1 pulse/s. After a 30-min pause, five more pulses with the same parameters were delivered. To determine the effect of Nmnat3 overexpression on axonal degeneration, intravitreal injection of TNF was performed 1 day after ELP, or the hypertensive glaucoma model was generated 6 h after ELP with or without intravitreal injection of 3-MA (2-*μ*l; 60 mM in DMSO; Sigma-Aldrich). Transfection of the Nmnat3–EGFP plasmid in the retina and optic nerve was confirmed with immunoblotting using Nmnat3 antibody. Flat-mounted retinas were also examined to confirm the transfection of the Nmnat3–EGFP plasmid under a confocal microscope. Retrograde Fluoro-gold (Fluorochrome) labeling of RGCs was performed 1 week before transfection with EGFP–Nmnat3 as described previously.^14^ Transverse-sectioned retinas 1 week after transfection with EGFP–Nmnat3 were also examined to confirm the transfection.

### Electron microscopy

Eyes were obtained from animals 3 weeks after IOP elevation. Approximately 8-mm segments of the optic nerves were sampled starting around the optic nerve head to examine both unmyelinated and myelinated areas. These segments of optic nerve were fixed by immersion in Karnovsky's solution for 24 h at 4 °C, processed, and embedded in acrylic resin. Ultrathin sections (∼100 nm thick) were prepared using an LKB Ultrotome V (LKB-Produkter AB, Bromma, Sweden), contrasted with saturated aqueous uranyl acetate and Sato's lead solution, and viewed with a JEOL 1200EX transmission electron microscope (JEOL, Tokyo, Japan) at 80 kV.

### Axon counting in optic nerves

Morphometric analysis of each optic nerve was performed as described previously with samples from 18 rats (TNF group) and 30 rats (glaucoma group).^25, 45^ Eyes were obtained from the animals 2 weeks after intravitreal injection (TNF group) or 3 weeks after IOP elevation (glaucoma group). Four-millimeter segments of the optic nerves were obtained starting 1 mm behind the globe. These segments of optic nerve were fixed by immersion in Karnovsky's solution for 24 h at 4 °C, processed, and embedded in acrylic resin. Cross-sections (1 *μ*m thick) were cut beginning 1 mm from the globe and stained with a solution of 1% paraphenylene-diamine (Sigma-Aldrich) in absolute methanol. For each section, images at the center and at each quadrant of the periphery (∼141.4 *μ*m from the center) were acquired with a light microscope (BX51; Olympus, Tokyo, Japan) with a × 100 coupled digital camera (MP5Mc/OL; Olympus) and associated QCapture Pro software (version 5.1, QImaging, Surrey, British Columbia, Canada). The acquired images were quantified using Aphelion image processing software (version 3.2, ADCIS SA and AAI, Inc., Hérouville Saint Clair, France). The number of axons was determined in five distinct areas of 1446.5 *μ*m^2^ each (each quadrant of the periphery in addition to the center; total area of 7232.3 *μ*m^2^ per eye) from each eye. The number of axons per eye was averaged and expressed as number per square millimeter. A minimum of four eyes per experimental condition was used for analysis.

### Expression of p62 and LC3 turnover assay in RGC-5 cells

RGC-5 cells were maintained in Dulbecco's modified Eagle's medium (DMEM) containing 10% fetal bovine serum (Sigma-Aldrich), 100 U/ml penicillin, and 100 *μ*g/ml streptomycin (Sigma-Aldrich) in a humidified atmosphere of 95% air and 5% CO_2_ at 37 °C, as described previously.^25, 45^ For subcellular localization, RGC-5 cells were seeded on a collagen-coated plate, grown to 80% confluence, and incubated in the above DMEM or serum-free DMEM for 24 h.^2^ Cells were incubated with 500 nM MitoTracker Red CMXRox (Invitrogen) for 30 min at 37 °C, fixed with 4% PFA for 30 min, and washed three times with T-PBS. After blocking with 5% BSA, cells were incubated with primary antibodies for Nmnat3 (1 : 100; ProSci Incorporated) or p62 (1 : 200; Medical & Biological Laboratories Co.). The cells were incubated with FITC-labeled anti-goat antibody (1 : 100; Cappel, MP Biomedicals, LLC.), FITC-labeled anti-rabbit antibody (1 : 100; Cappel, MP Biomedicals, LLC.), or rhodamine-labeled anti-rabbit antibody (1 : 100; Cappel, MP Biomedicals, LLC.). The cells were counterstained with DAPI (Vectashield with DAPI, Vector Laboratories, Inc.). Images were acquired with a confocal microscope. Quantification of p62 puncta was performed as described previously.^46^ For LC3 turnover assay, a lipofectamine-mediated transfection procedure was used to introduce Nmnat3-expressing plasmid DNA into the RGC-5 cells.^25^ The cells were also treated with or without 20 *μ*M chloroquine (Sigma-Aldrich).^41^ At 24 h after transfection, cells were collected and used for immunoblot analysis and quantitative reverse-transcription PCR (Light-Cycler; Roche Diagnostics, Tokyo, Japan), as described previously.^19^ Total RNA was isolated using the ReliaPrep RNA Cell Miniprep System (Promega Corporation, Madison, WI, USA). The primers for p62 were 5′-GCTGCCCTATACCCACATCT-3′ (sense) and 5′-CGCCTTCATCCGAGAAAC-3′ (antisense).^47^ The primers for 18S ribosomal RNA were 5′-AAGTTTCAGCACATCCTGCGAGTA-3′ (sense) and 5′-TTGGTGAGGTCAATGTCTGCTTTC-3′ (antisense).

### Statistical analysis

Data are presented as mean ± S.E.M. Differences among groups were analyzed using one-way ANOVA, followed by Scheffe's method or Mann–Whitney's method. A probability value of <0.05 was considered to represent a statistically significant difference.

## Abbreviations

Nmnat: nicotinamide mononucleotide adenylyltransferase
IOP: intraocular pressure
LC3: microtubule-associated protein light chain 3
RGC: retinal ganglion cell
TNF: tumor necrosis factor
DRG: dorsal root ganglia
GFP: green fluorescent protein
ELP: electroporation
PBS: phosphate-buffered saline
3-MA: 3-methyladenine
Trx2: thioredoxin 2
NAD: nicotinamide adenine dinucleotide
ROS: reactive oxygen species
DAPI: 4′,6-diamidino-2-phenylindole
DMSO: dimethyl sulfoxide
TBS: Tris-buffered saline
DMEM: Dulbecco's modified Eagle's medium
