# Supplementary figures and images for: Axonal protection by Nmnat3 overexpression with involvement of autophagy in optic nerve degeneration
Source: Cell Death Dis. 2013 Oct 17;4(10):e860–. doi: 10.1038/cddis.2013.391 (PMC3920931; doi:10.1038/cddis.2013.391)

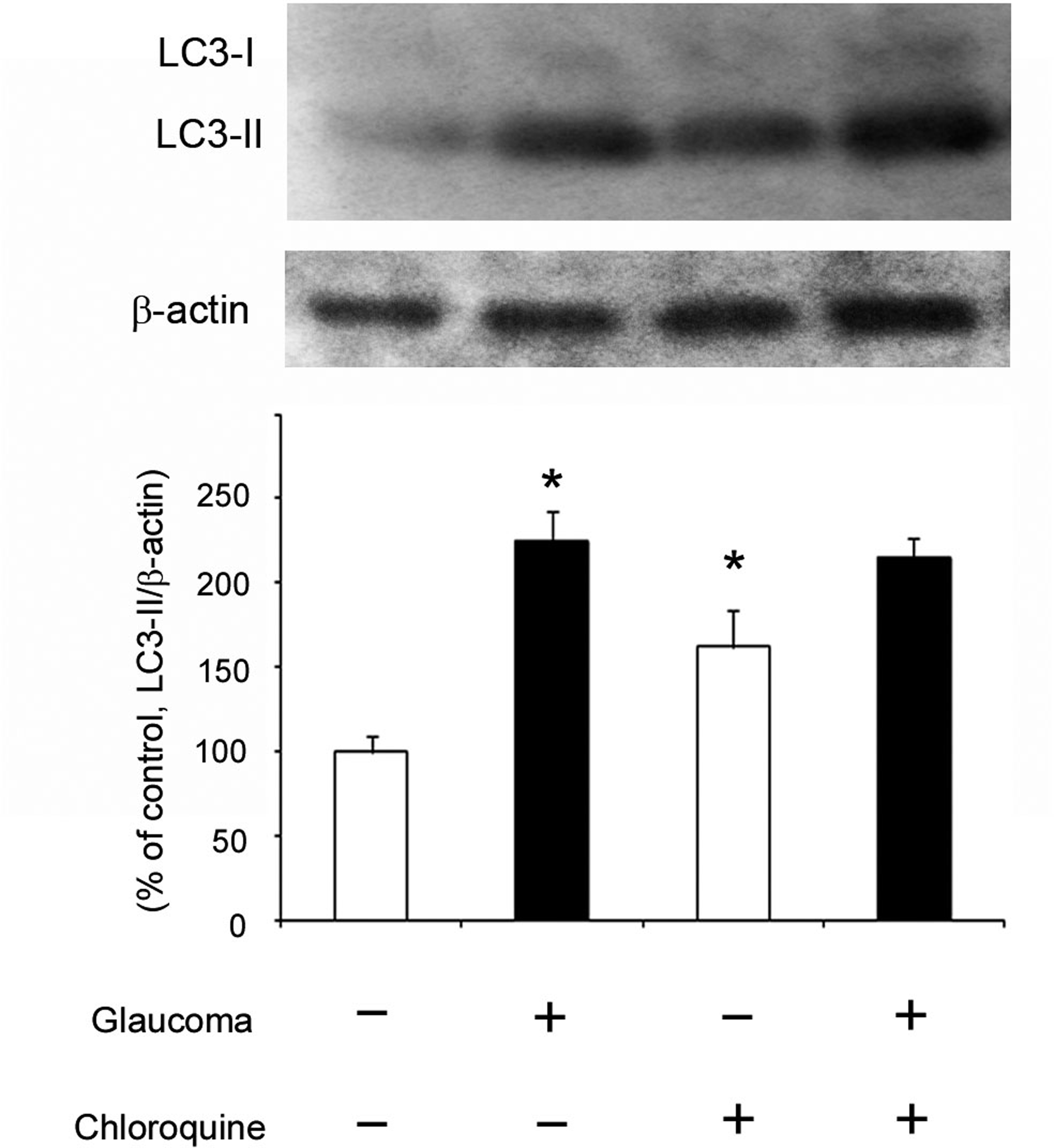

Supplement: Supplementary Figure 1 [file cddis2013391x1.tif]
