# Supplementary material for: Axonal protection by Nmnat3 overexpression with involvement of autophagy in optic nerve degeneration
Source: Cell Death Dis. 2013 Oct 17;4(10):e860–. doi: 10.1038/cddis.2013.391 (PMC3920931; doi:10.1038/cddis.2013.391)
Supplement: Supplementary Information [file cddis2013391x2.doc]

**Supplementary Figure legends**

**Supplementary Figure 1** Effect of chloroquine on LC-3 II levels in optic nerves. The difference in LC-3 II levels between optic nerves with and without chloroquine (60 mg/kg, i.p.) treatment 12 h before enucleation of the eye was compared under control conditions and 1 week after glaucoma induction. Data are normalized to β-actin levels in the same sample. Data are expressed as a percentage of control. Each column represents mean ± SEM; *n* = 4 per group. *p < 0.05 versus control.
